# Editorial: Investigating lung biology, metabolism, and host susceptibility in mycobacterial infections

**DOI:** 10.3389/fcimb.2026.1869137

**Published:** 2026-05-21

**Authors:** Pallavi Chandra, Andrew T. Roth, Katharina Ronacher

**Affiliations:** 1 Division of Infectious Diseases, Department of Medicine, Washington University School of Medicine, St. Louis, MO, United States; 2 Division of Pulmonary and Critical Care Medicine, Department of Medicine, Washington University School of Medicine, St. Louis, MO, United States; 3 Australian Infectious Diseases Research Centre, The University of Queensland, Brisbane, QLD, Australia; 4 Translational Research Institute, Mater Research Institute, Faculty of Health, Medicine and Behavioural Sciences, The University of Queensland, Brisbane, QLD, Australia

**Keywords:** granuloma, host susceptibility, lung biology, macrophage, mycobacteria, Mycobacterium avium, tuberculosis

*Mycobacterium tuberculosis* and nontuberculous mycobacteria (NTM) are a substantial source of global morbidity and mortality. *M. tuberculosis* causes tuberculosis (TB) which kills more than one million people annually, while infections due to NTM are increasing in incidence and mortality worldwide (Harada et al., 2025; W.H.O., 2025). Mycobacteria have a distinct cell wall structure and conserved virulence mechanisms that distinguish them from other bacterial infections. Following infection of macrophages, they survive intracellularly by disrupting vesicular trafficking and influencing host cell death pathways. They reprogram macrophage metabolism, driving the formation of lipid overloaded and dysfunctional “foamy macrophages” (Singh et al., 2012). The host initially contains the bacteria within organized immune structures called granulomas; however, these structures can become vehicles for pathogen progression (Lyu et al., 2024). Mycobacteria also modulate host inflammatory pathways, evading inflammatory bactericidal programs while promoting inflammation that can result in structural lung disease, including fibrosis (Faysal et al., 2025). In this Research Topic, we present original research and reviews that dissect important host-pathogen interactions underlying mycobacterial infections.

Kajiwara et al. investigated the role of the protein Apoptosis Inhibitor of Macrophage (AIM/CD5L) in *M. avium* lung infection. AIM is produced by tissue-resident macrophages and promotes survival and phagocytic clearance of debris (Arai et al., 2005; Sanjurjo et al., 2015). Previous work by the authors found that AIM accumulated in the lungs during *M. avium* infection where it was associated with the development of foamy macrophages (Kajiwara et al., 2023). Here, the authors utilized AIM deficient (*Aim-/-*) mice to understand how this protein increases susceptibility to infection. Following infection with *M. avium* intratracheally, *Aim^-/-^* mice had reduced lung bacterial burden and immunopathology in the lungs relative to wild-type animals. *Aim^-/-^* mice showed reduced expression of genes involved in lipid droplet synthesis (*Acsl6*, *Dgat1*, *Acat1*) coupled with increased expression of the cholesterol efflux pump *Abca1*, demonstrating the role of AIM in macrophage metabolism. Using flow cytometry, the authors showed that *Aim^-/-^* mice had increased activated macrophages and interferon-γ producing T cells and lower IL-10 concentrations, which they hypothesized were responsible for enhanced bacterial control.

Ma et al. provided a thorough review of PANoptosis, presenting early evidence that this may be an important mechanism of cell death in response to *M. tuberculosis*. PANoptosis is a distinct inflammatory cell death pathway where pyroptotic, apoptotic, and necroptotic machinery are activated through multiprotein PANoptosomes (Malireddi et al., 2019). While all three of these cell death pathways have established roles in mycobacterial pathogenesis and PANoptosis has been demonstrated in other intracellular bacterial pathogens including *Yersinia*, direct evidence of PANoptosis in response to *M. tuberculosis* is limited (Malireddi et al., 2020). The authors note that the upstream regulators of PANoptosis, AIM2 and ZBP1, are upregulated in multiple TB transcriptional datasets. Furthermore, ZBP1 and several PANoptosome components were found to be increased in macrophages treated with an attenuated strain of *M. tuberculosis (*Shen et al., 2025*)*. The authors lay out priorities to test their hypotheses directly in *M. tuberculosis*.

Seidl et al. analyzed lymph node biopsies from 33 children with NTM cervical lymphadenitis to determine how pathological features are associated with clinical outcomes. Germinal centers, increased CD8+ T cell density within necrotic areas, and foamy epithelioid cells were associated with favorable clinical outcomes while CD4+ T cell clustering, liquefaction on imaging, and skin changes were associated with complicated courses and unfavorable clinical outcomes. The authors provide a framework for using pathological features from lymph node biopsies to guide surgical versus conservative management of NTM lymphadenitis. Furthermore, this article builds on recent discoveries about how immune organization within granulomas contributes to control of mycobacteria (Chai et al., 2026; Doratt et al., 2025).

Chávez-Domínguez et al. reviewed the complex relationship between TB and lung cancer. The authors begin by reviewing epidemiological data which have consistently demonstrated the relationship between TB and lung cancer across diverse populations (Swami et al., 2026; Wu et al., 2011). Additionally, sites of TB-associated lung damage including old granulomas and fibrotic scars are frequent sites of lung cancers (Yu et al., 2008). Chávez-Domínguez et al. discuss how complex host-pathogen interactions including production of cytokines and reactive oxygen species, DNA damage, angiogenesis, and alterations in immune checkpoints may underly the oncogenic transformation of epithelial cells. They conclude by discussing how candidate biomarkers and other future strategies can be used to identify individuals with TB-associated lung disease who are at the highest risk for developing lung cancer.

## Conclusion

These articles address key aspects of host–mycobacteria interactions: metabolic reprogramming of macrophages and integration of cell death pathways as determinants of host control of infection; granulomas as both protective structures and staging grounds for infection progression; and inflammation as a cause of structural lung disease and oncogenesis. We hope these contributions will spur further investigation and increase our understanding of these challenging infections.

## References

[B1] Arai S. Shelton J. M. Chen M. Bradley M. N. Castrillo A. Bookout A. L. . (2005). A role for the apoptosis inhibitory factor AIM/Spalpha/Api6 in atherosclerosis development. Cell Metab. 1, 201–213. doi: 10.1016/j.cmet.2005.02.002. PMID: 16054063

[B2] Chai Q. Lu Z. Zhao M. Yu S. Zhong Y. Qiu C. . (2026). Lipid accumulation in tuberculosis granulomas inhibits macrophage-CD4(+) T cell interactions and infection control. Nat. Microbiol. doi: 10.1038/s41564-026-02317-3. PMID: 41933199

[B3] Doratt B. M. Napier E. G. Boroujeni M. E. Douglas S. Davies M. H. Bermudez L. . (2025). Granulomatous cellular signatures in nontuberculous and tuberculous mycobacterial infections. Front. Microbiol. 16, 1741883. doi: 10.3389/fmicb.2025.1741883. PMID: 41586350 PMC12823825

[B4] Faysal M. A. Hanafy M. Zinniel D. K. Tanni F. Y. Muthukrishnan E. Rathnaiah G. . (2025). Cell death pathways in response to Mycobacterium tuberculosis and other mycobacterial infections. Infect. Immun. 93, e0040125. doi: 10.1128/iai.00401-25. PMID: 40924403 PMC12519786

[B5] Harada K. Vu Q. T. Nishimura Y. Takeda T. Hamano H. Minato Y. . (2025). Trends in nontuberculous mycobacterial disease mortality based on 2000–2022 data from 83 countries. Int. J. Infect. Dis. 158, 107932. doi: 10.1016/j.ijid.2025.107932. PMID: 40354928

[B6] Kajiwara C. Shiozawa A. Urabe N. Yamaguchi T. Kimura S. Akasaka Y. . (2023). Apoptosis inhibitor of macrophages contributes to the chronicity of mycobacterium avium infection by promoting foamy macrophage formation. J. Immunol. 210, 431–441. doi: 10.4049/jimmunol.2200306. PMID: 36602769

[B7] Lyu J. Narum D. E. Baldwin S. L. Larsen S. E. Bai X. Griffith D. E. . (2024). Understanding the development of tuberculous granulomas: insights into host protection and pathogenesis, a review in humans and animals. Front. Immunol. 15, 1427559. doi: 10.3389/fimmu.2024.1427559. PMID: 39717773 PMC11663721

[B8] Malireddi R. K. S. Kesavardhana S. Kanneganti T. D. (2019). ZBP1 and TAK1: master regulators of NLRP3 inflammasome/pyroptosis, apoptosis, and necroptosis (PAN-optosis). Front. Cell. Infect. Microbiol. 9, 406. doi: 10.3389/fcimb.2019.00406. PMID: 31850239 PMC6902032

[B9] Malireddi R. K. S. Kesavardhana S. Karki R. Kancharana B. Burton A. R. Kanneganti T. D. (2020). RIPK1 distinctly regulates yersinia-induced inflammatory cell death, PANoptosis. Immunohorizons 4, 789–796. doi: 10.4049/immunohorizons.2000097. PMID: 33310881 PMC7906112

[B10] Sanjurjo L. Amezaga N. Aran G. Naranjo-Gomez M. Arias L. Armengol C. . (2015). The human CD5L/AIM-CD36 axis: A novel autophagy inducer in macrophages that modulates inflammatory responses. Autophagy 11, 487–502. doi: 10.1080/15548627.2015.1017183. PMID: 25713983 PMC4502645

[B11] Shen J. Fu Y. Liu F. Wu J. Zhang H. Sun J. . (2025). Salvianolic acid B inhibits ZBP1-mediated PANoptosis in mycobacterium tuberculosis-infected macrophages by targeting TNFR1. Phytother. Res. 39, 4028–4045. doi: 10.1002/ptr.70042. PMID: 40717039

[B12] Singh V. Jamwal S. Jain R. Verma P. Gokhale R. Rao K. V. (2012). Mycobacterium tuberculosis-driven targeted recalibration of macrophage lipid homeostasis promotes the foamy phenotype. Cell Host Microbe 12, 669–681. doi: 10.1016/j.chom.2012.09.012. PMID: 23159056

[B13] SwamiN. HongJ. H. KhoS. KangH. M. Chun-Chia LinL. NiS. C. . (2026). Associations between prior lung diseases and risk of lung cancer in populations with no smoking history: A systematic review and meta-analysis. Chest. doi: 10.1016/j.chest.2025.12.034. PMID: 41534708

[B14] W.H.O (2025). Global tuberculosis report 2025 ( World Health Organization).

[B15] Wu C. Y. Hu H. Y. Pu C. Y. Huang N. Shen H. C. Li C. P. . (2011). Pulmonary tuberculosis increases the risk of lung cancer: a population-based cohort study. Cancer 117, 618–624. doi: 10.1002/cncr.25616. PMID: 20886634

[B16] Yu Y. Y. Pinsky P. F. Caporaso N. E. Chatterjee N. Baumgarten M. Langenberg P. Furuno J. P. Lan Q. Engels E. A. (2008). Lung cancer risk following detection of pulmonary scarring by chest radiography in the prostate, lung, colorectal, and ovarian cancer screening trial. Arch Intern Med. 168 (21), 2326–2332. doi: 10.1001/archinte.168.21.2326 19029496 PMC2866505

